# Inhibition of Skin Inflammation by Scytonemin, an Ultraviolet Sunscreen Pigment

**DOI:** 10.3390/md18060300

**Published:** 2020-06-04

**Authors:** Moo Rim Kang, Sun Ah Jo, Hyunju Lee, Yeo Dae Yoon, Joo-Hee Kwon, Jeong-Wook Yang, Byeong Jo Choi, Ki Hwan Park, Myeong Youl Lee, Chang Woo Lee, Kyeong-Ryoon Lee, Jong Soon Kang

**Affiliations:** 1 Laboratory Animal Resource Center, Korea Research Institute of Bioscience and Biotechnology, 30 Yeongudanjiro, Cheongju 28116, Korea; kangmr@ractigen.com (M.R.K.); jsunada123@naver.com (S.A.J.); hyunju35@kribb.re.kr (H.L.); yunyd76@kribb.re.kr (Y.D.Y.); juhee@kribb.re.kr (J.-H.K.); z7V8@kribb.re.kr (J.-W.Y.); byung127@kribb.re.kr (B.J.C.); brightnessd@nate.com (K.H.P.); myong@kribb.re.kr (M.Y.L.); changwoo@kribb.re.kr (C.W.L.); kyeongrlee@kribb.re.kr (K.-R.L.); 2 Ractigen Therapeutics, Nantong, Jiangsu 226400, China

**Keywords:** scytonemin, skin inflammation, tumor necrosis factor-α, nitric oxide, NF-κB

## Abstract

Scytonemin is a yellow-green ultraviolet sunscreen pigment present in different genera of aquatic and terrestrial blue-green algae, including marine cyanobacteria. In the present study, the anti-inflammatory activities of scytonemin were evaluated in vitro and in vivo. Topical application of scytonemin inhibited 12-*O*-tetradecanoylphorbol-13-acetate (TPA)-induced ear swelling in BALB/c mice. The expression of tumor necrosis factor-α (TNF-α) and inducible nitric oxide synthase (iNOS) was also suppressed by scytonemin treatment in the TPA-treated ear of BALB/c mice. In addition, scytonemin inhibited lipopolysaccharide (LPS)-induced production of TNF-α and nitric oxide (NO) in RAW 264.7 cells, a murine macrophage-like cell line, and the mRNA expressions of TNF-α and iNOS were also suppressed by scytonemin in LPS-stimulated RAW 264.7 cells. Further study demonstrated that LPS-induced NF-κB activity was significantly suppressed by scytonemin treatment in RAW 264.7 cells. Our results also showed that the degradation of IκBα and nuclear translocation of the p65 subunit were blocked by scytonemin in LPS-stimulated RAW 264.7 cells. Collectively, these results suggest that scytonemin inhibits skin inflammation by blocking the expression of inflammatory mediators, and the anti-inflammatory effect of scytonemin is mediated, at least in part, by down-regulation of NF-κB activity. Our results also suggest that scytonemin might be used as a multi-function skin care ingredient for UV protection and anti-inflammation.

## 1. Introduction

**Figure 1:**
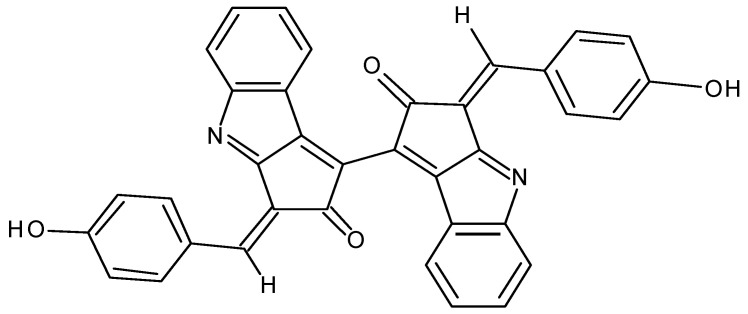
Chemical structure of scytonemin.

Recently, cyanobacterial metabolites called attention to their biotechnological and industrial significance and have been exploited by pharmaceutical and cosmetic industries. Scytonemin, a cyanobacterial metabolite, is a small hydrophobic pigment molecule present in the extracellular sheath of certain strains of cyanobacteria and has been known to exert a protective role against solar ultraviolet (UV) radiation [1]. This pigment is a dimeric molecule of indolic and phenolic subunits (Figure 1) and has the ability to reduce the production of reactive oxygen species and the formation of DNA lesions [1,2]. Scytonemin has also been reported to exert a variety of biological activities, including antioxidant and anti-proliferative activities [3,4].

Skin inflammation is a physiological reaction to tissue injury, pathogen invasion, and irritants, and occurs due to the immune response to these insults [5]. Innate and/or adaptive immune cells are recruited to the site of inflammation and play a vital role in the host defense against pathogens by initiation and propagation of inflammatory responses [6]. However, excessive or persistent inflammation causes a variety of pathological conditions, such as skin inflammation and rheumatoid arthritis [7]. Macrophages are the primary pro-inflammatory cells, and the activation of macrophages mediate inflammatory responses by the production of inflammatory mediators, such as tumor necrosis factor-α (TNF-α) and nitric oxide (NO) [8,9]. TNF-α is the most well-known pro-inflammatory cytokine and implicated in various immune and inflammatory responses [10]. In inflammatory conditions, inducible nitric oxide synthase (iNOS) is expressed by macrophages and produces a large amount of NO, which mediates a variety of biological responses, including antimicrobial defense and antitumor activity [11,12]. The transcriptional regulation of TNF-α and iNOS is a tightly controlled event, and NF-κB is a major transcription factor involved in the expression of these inflammatory mediators [13,14]. In this study, we examined the inhibitory effect of scytonemin on skin inflammation in mice and confirmed the anti-inflammatory effect of scytonemin in mouse macrophages. We also briefly investigated the mechanisms responsible for the anti-inflammatory effect of scytonemin.

## 2. Results

### 2.1. Effect of Scytonemin on 12-O-tetradecanoylphorbol-13-acetate (TPA)-Induced Ear Swelling in BALB/c Mice

**Figure 2:**
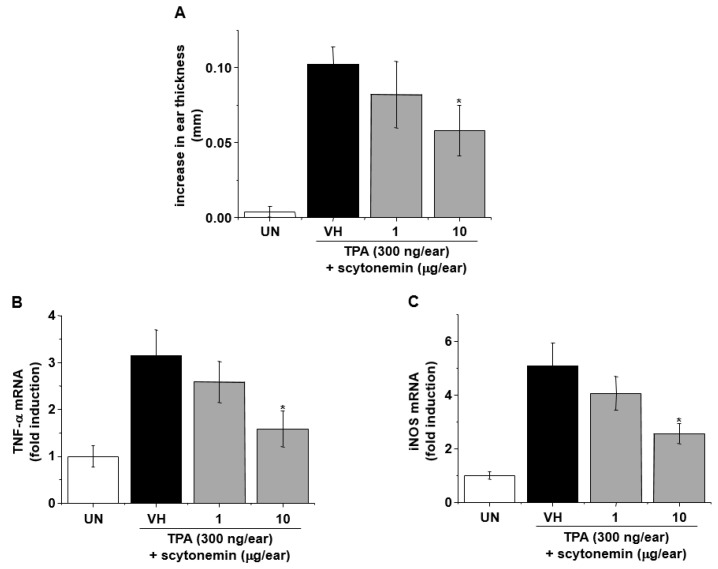
Effect of topical application of scytonemin on 12-*O*-tetradecanoylphorbol-13-acetate (TPA)-induced skin inflammation. (**A**) The ears of BALB/c mice were pretreated with vehicle or the indicated concentrations of scytonemin (1 or 10 μg/ear) for 30 min, and TPA (300 ng/ear, dissolved in acetone:olive oil = 4:1 (AOO)) was applied to induce skin inflammation. After 4 h, the increase in ear thickness was measured. Each column shows the mean ± SD (*n* = 5). Total RNAs were isolated from ear tissues, and mRNA expression of iNOS (**B**), TNF-α (**C**), and β-actin were determined by RT-PCR. Each column shows the ± SD of triplicate determinations. Significance was determined using Dunnett’s *t*-test versus the control group (* *p <* 0.05).

To investigate the anti-inflammatory effect of scytonemin in vivo, we examined the effect of topically applied scytonemin on TPA-induced ear swelling in BALB/c mice. Topical application of TPA (300 ng/ear) induced ear swelling after 4 h in BALB/c mice, and TPA-induced increase in ear thickness was significantly suppressed by scytonemin treatment (Figure 2A). To further investigate, we also examined the effect of topically applied scytonemin on TPA-induced gene expression of inflammatory mediators using ear biopsies. As shown in Figure 2B,C, scytonemin significantly down-regulated the gene expression of TNF-α and iNOS in the TPA-treated ear of BALB/c mice.

### 2.2. Effect of Scytonemin on Lipopolysaccharide (LPS)-induced Production of Inflammatory Mediators in RAW 264.7 Cells

**Figure 3:**
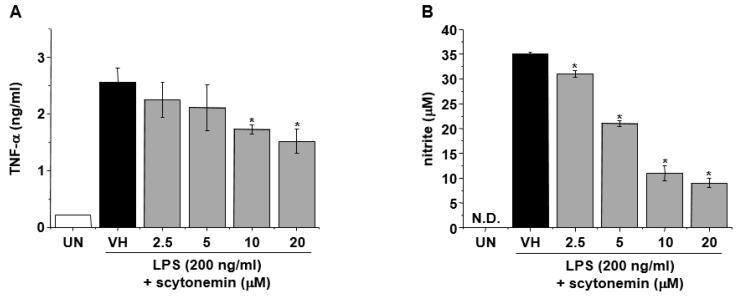
Effect of scytonemin on the production of TNF-α and nitrite in LPS-stimulated RAW 264.7 cells. RAW 264.7 cells were pretreated with the indicated concentrations of scytonemin for 1 h before being incubated with LPS (200 ng/mL) for 24 h. The culture supernatants were collected, and the levels of TNF-α (**A**) and nitrite (**B**) were measured. Each column shows the mean ± SD of triplicate determinations. Significance was determined using Dunnett’s *t*-test versus the control group (* *p <* 0.05). ND = not detected.

To further confirm the anti-inflammatory effect of scytonemin, we examined the effect of scytonemin on the production of inflammatory mediators in mouse macrophage cell line, RAW 264.7. Figure 3A shows that the secretion of TNF-α was significantly increased by LPS (200 ng/mL) treatment in RAW 264.7 cells, and this was dramatically inhibited by scytonemin treatment in a dose-dependent manner. In addition, the treatment of RAW 264.7 cells with LPS also increased the accumulation of nitrite and scytonemin dose-dependently suppressed the LPS-induced accumulation of nitrite (Figure 3B). Treatment with 20 μM of scytonemin caused 40.4% and 74.3% inhibition of TNF-α and NO production, respectively, in LPS-stimulated RAW 264.7 cells (Figure 3A,B). Scytonemin had no significant cytotoxic effects in LPS-stimulated RAW 264.7 cells at concentrations used in this study (Supplementary Figure S1).

### 2.3. Effect of Scytonemin on LPS-induced mRNA Expression of Inflammatory Mediators in RAW 264.7 Cells

**Figure 4:**
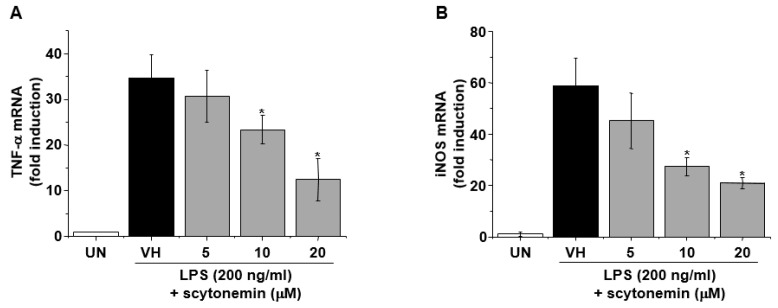
Effect of scytonemin on the mRNA expression of TNF-α and iNOS in LPS-stimulated RAW 264.7 cells. RAW 264.7 cells were pretreated with the indicated concentrations of scytonemin for 1 h before being incubated with LPS (200 ng/mL) for 6 h. Total RNAs were isolated, and TNF-α (**A**) and iNOS (**B**) mRNA expression was determined by RT-PCR. Each column shows the mean ± SD of triplicate determinations. Significance was determined using Dunnett’s *t*-test versus the control group (* *p <* 0.05).

To further investigate whether the inhibitory effect of scytonemin on the production of TNF-α and NO in LPS-stimulated RAW 264.7 cells are due to the inhibitory effect of scytonemin on the mRNA expression of cognate genes, the effect of scytonemin on LPS-induced mRNA expressions of TNF-α and iNOS were assessed by quantitative RT-PCR. As shown in Figure 4A,B, LPS markedly induced the mRNA levels of TNF-α and iNOS, and this induction was dose-dependently inhibited by scytonemin treatment in RAW 264.7 cells.

### 2.4. Effect of Scytonemin on LPS-induced NF-κB Signaling in RAW 264.7 Cells

**Figure 5:**
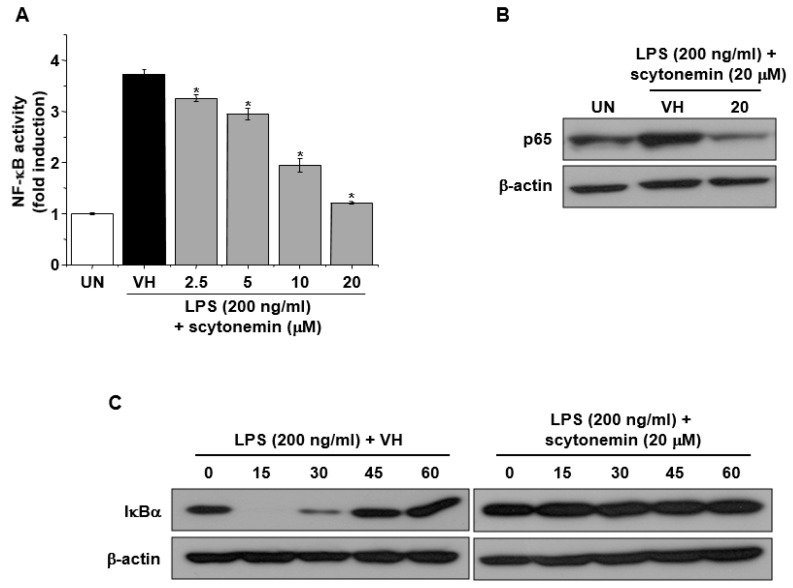
Effect of scytonemin on NF-κB activation, p65 nuclear translocation, and IκBα degradation in LPS-stimulated RAW 264.7 cells. (**A**) RAW 264.7 cells were transiently transfected with pNF-κB-Luc containing five copies of the NF-κB/Rel binding site, treated with the indicated concentrations of scytonemin, and LPS (200 ng/mL) for 24 h, and assayed for luciferase expression using a luciferase assay kit. (**B**) RAW 264.7 cells were pretreated with the indicated concentrations of scytonemin for 1 h, incubated with LPS (200 ng/mL) for 30 min, and then assayed for the nuclear translocation of p65 by Western immunoblot analysis. (**C**) RAW 264.7 cells were pretreated with 20 μM of scytonemin for 1 h, incubated with LPS (200 ng/mL) for the indicated times, and then assayed for the degradation of IκBα by Western immunoblot analysis.

To investigate the molecular mechanisms responsible for the inhibitory effect of scytonemin on the gene expression of TNF-α and iNOS, we examined the effect of scytonemin on NF-κB activity in LPS-stimulated 264.7 cells. As shown in Figure 5A, LPS treatment caused a marked increase in NF-κB activity in RAW 264.7 cells. However, scytonemin treatment suppressed LPS-induced NF-κB activity in a dose-dependent manner (Figure 5A). Figure 5B also shows that scytonemin suppressed the nuclear translocation of p65 in LPS-stimulated RAW 264.7 cells. Moreover, our results also showed that LPS-induced degradation of IκBα was blocked by scytonemin treatment in RAW 264.7 cells (Figure 5C).

## 3. Discussion

Scytonemin is well-known as a potent UV sunscreen agent, and it has been suggested that scytonemin can be exploited for the development of cosmeceuticals [1]. Among various functional properties of cosmetic products, anti-inflammation is one of the most important properties along with UV protection, whitening, and anti-wrinkle properties. In this study, we demonstrated that scytonemin possesses an anti-inflammatory property and inhibits inflammation in vitro and in vivo, which suggests that scytonemin might serve a dual function as a topically applicable ingredient for both UV protection and anti-inflammation.

Skin provides a mechanical and immunological barrier between the body and the environment, and dysregulated inflammatory reactions in the skin can cause a variety of skin diseases [15]. Exacerbated inflammation is a hallmark of a variety of inflammatory skin diseases, including dermatitis, psoriasis, and rosacea [16]. Skin inflammation is also known as a key process involved in tumorigenesis and wound healing in the skin [17,18]. Therefore, it is required to down-regulate exacerbated or persistent inflammation of the skin to prevent these pathological conditions. In this study, we investigated the anti-inflammatory effect of scytonemin in a mouse model of skin inflammation. Here, we clearly demonstrated that topical application of mouse ear with TPA caused skin inflammation, which was manifested by edema and erythema. However, treatment with scytonemin significantly inhibited ear swelling induced by TPA, suggesting that topically applied scytonemin exerts an anti-inflammatory effect in vivo.

TNF-α and nitric oxide are well-known pro-inflammatory mediators, and the expression of TNF-α and iNOS is increased in inflamed tissue. The role of TNF-α and nitric oxide in skin inflammation has been well documented previously [19,20,21]. Therefore, we investigated the effect of scytonemin on the expression of TNF-α and iNOS in TPA-treated mouse ears. Our results showed that TPA-induced expression of TNF-α and iNOS was significantly suppressed by scytonemin treatment. To further confirm, we also examined the effect of scytonemin on the production and mRNA expression of these inflammatory mediators in mouse macrophage cell line, RAW 264.7. Consistent with the results of in vivo experiments, scytonemin dose-dependently inhibited the production of TNF-α and nitric oxide in LPS-stimulated RAW 264.7 cells. In addition, the mRNA expression of TNF-α and iNOS was also suppressed by scytonemin treatment, suggesting that the decreased production of TNF-α and nitric oxide might be due to the decreased mRNA expression of cognate genes.

The mRNA expression of TNF-α and iNOS is regulated at both transcriptional and post-transcriptional level, and the transcriptional regulation of these genes are a tightly controlled event [22]. Among various transcription factors, NF-κB is a major transcription factor involved in the transcriptional regulation of these inflammatory mediators [13,14]. NF-κB is a pleiotropic regulator of various genes involved in immune and inflammatory responses. In unstimulated cells, NF-κB exists in an inactive state, in the cytoplasm, complexed with the inhibitory protein, called IκB. Upon activation, IκB undergoes phosphorylation and degradation, and the NF-κB, including p65, is translocated into the nucleus, where it binds to DNA and activates transcription [23]. To elucidate the molecular mechanism responsible for the inhibitory effect of scytonemin on the mRNA expression of TNF-α and iNOS, we examined the effect of scytonemin on NF-κB activity. Transient transfection and reporter gene assay revealed that LPS-induced NF-κB activity was significantly suppressed by scytonemin treatment in RAW 264.7 cells, suggesting that the inhibitory effect of scytonemin might be mediated by blocking the NF-κB activity.

## 4. Materials and Methods 

### 4.1. Chemicals, Animals and Cell Culture

All reagents were purchased from Sigma-Aldrich (St Louis, MO, USA) unless otherwise stated. Scytonemin was purchased from Alexis Biochemicals (San Diego, CA, USA). Female BALB/c mice (5 weeks old) were purchased from Koatech (Pyungtaek, Gyeonggi, Korea) and cared for as described previously [24]. RAW 264.7 cells (ATCC TIB71) were grown in Dulbecco’s modified Eagle’s medium (Invitrogen, Carlsbad, CA, USA), supplemented with 10% fetal bovine serum, 2 mM l-glutamine, 100 U/mL penicillin, and 100 mg/mL streptomycin at 37 °C in 5% CO_2_ humidified air.

### 4.2. TPA-induced Skin Inflammation

Skin inflammation was induced by topical application of 20 μL of TPA (15 μg/mL in acetone:olive oil = 4:1 (AOO)) to each ear of the BALB/c mice. The indicated concentrations of scytonemin (1 or 10 μg/ear, dissolved in AOO) were treated 30 min before TPA treatment. Ear thickness was measured using a digital thickness gauge (Digimatic Indicator, Mitsutoyo, Tokyo, Japan) just before and 4 h after TPA treatment, and the ear swelling was determined by subtracting the ear thickness at 0 h from that at 4 h.

### 4.3. Total RNA Isolation and Quantification of mRNA Expression

Total RNA isolation and quantitative RT-PCR were performed as described previously [25] with slight modifications. Briefly, RNA was isolated using TRIzol reagent (Invitrogen, Carlsbad, CA, USA) as described previously [26]. Equal amounts of RNA were reverse transcribed into cDNA by using oligo(dT)_15_ primers. qPCR was performed using the Power SYBR Green PCR Master Mix (Invitrogen, Carlsbad, CA, USA). Samples were amplified by 40 cycles of denaturation (95 °C for 15 s), annealing (56 °C for 30 s), and extension (72 °C for 45 s) using ABI 7500 Sequence Detection System (Applied Biosciences, Foster City, CA, USA). The primer sequences used were as follows: mouse iNOS, sense 5’-CTG CAG CAC TTG GAT CAG GAA CCT G-3’, antisense 5’-GGG AGT AGC CTG TGT GCA CCT GGA A-3’; mouse TNF-α, sense 5’-CCT GTA GCC CAC GTC GTA GC-3’, antisense 5’-TTG ACC TCA GCG CTG AGT TG-3’; mouse β-actin, sense 5’-TGG AAT CCT GTG GCA TCC ATG AAA C-3’, antisense 5’-TAA AAC GCA GCT CAG TAA CAG TCC G-3’.

### 4.4. Nitrite Quantification

NO_2_^−^ accumulation was used as an indicator of NO production in the medium, as described previously [12]. RAW 264.7 cells were plated at 5 × 10^5^ cells/mL and stimulated with LPS (200 ng/mL) in the presence or absence of scytonemin (2.5, 5, 10, or 20 μM) for 24 h. The isolated supernatants were mixed with an equal volume of Griess reagent (1% sulfanilamide, 0.1% naphthylethylenediamine dihydrochloride, and 2% phosphoric acid), and incubated at room temperature for 10 min. NaNO_2_ was used to generate a standard curve, and nitrite production was determined by measuring the optical density at 540 nm.

### 4.5. Enzyme-linked Immunosorbent Assay (ELISA)

RAW 264.7 cells were plated at 5 × 10^5^ cells/mL and stimulated with LPS (200 ng/mL) in the presence or absence of scytonemin (2.5, 5, 10, or 20 μM) for 6 h. The culture supernatants were collected, and the amount of TNF-α was determined by mouse TNF-α ELISA kit (R&D Systems, Minneapolis, MN, USA) according to the manufacturer’s instructions.

### 4.6. Transient Transfection and Luciferase Reporter Gene Assay

RAW 264.7 cells were plated at a density of 1 × 10^5^ cells per 24-well plate. After 24 h of growth to 90% confluence, the cells were transfected with pNF-κB-Luc plasmid (5× NF-κB; Stratagene, San Diego, CA, USA) using a mixture of plasmid and Lipofectamine PLUS in OPTI-MEM according to manufacturer’s specification (Invitrogen, Carlsbad, CA, USA). The transfected cells were treated with LPS (200 ng/mL) and indicated concentrations of scytonemin (2.5, 5, 10, or 20 μM) for 24 h, and lysed. Luciferase activity was measured by using the luciferase assay kit (Promega, Madison, WI, USA) according to the manufacturer’s instruction.

### 4.7. Western Immunoblot Analysis

Twenty micrograms of whole-cell lysates were separated by 10% sodium dodecyl sulfate-polyacrylamide gel electrophoresis and electrotransferred to a nitrocellulose membrane (Amersham Biosciences UK, Ltd., Little Chalfont, Buckinghamshire, UK). Each membrane was pre-incubated for 1 h at room temperature in Tris-buffered saline, pH 7.6, containing 0.05% Tween 20 and 5% nonfat milk. Each nitrocellulose membrane was incubated with specific antibodies against p65, IκBα, and β-actin (Cell Signaling Technology, Danvers, MA, USA). Immunoreactive bands were then detected by incubating with secondary IgG antibody conjugated with horseradish peroxidase and visualizing with enhanced chemiluminescence reagents (GE Healthcare, Chicago, IL, USA).

### 4.8. Statistical Analysis

The mean ± SD was determined for each treatment group in each experiment. Data were analyzed by analysis of variance and Dunnett’s multiple comparison test by using Prism (GraphPad Software Inc., La Jolla, San Diego, CA, USA) software. The criterion for statistical significance was set at *p* < 0.05.

## 5. Conclusions

Collectively, the results presented in this report demonstrated that scytonemin inhibits TPA-induced skin inflammation in mice, and this was accompanied by decreased expression of TNF-α, and iNOS in inflammatory skin. Our results also showed that scytonemin suppresses LPS-induced expression of TNF-α, and iNOS in mouse macrophage cell line, and this is mediated, at least in part, by down-regulation of NF-κB activity. Considering the fact that scytonemin is a UV sunscreen agent, our results suggest the possible application of scytonemin as a multi-function ingredient for skin care.

## Supplementary Materials

The following are available online at https://www.mdpi.com/1660-3397/18/6/300/s1, Figure S1: Effect of scytonemin on the cell viability of LPS-stumulated RAW 264.7 cells.
