# Supplementary material for: Inhibition of Skin Inflammation by Scytonemin, an Ultraviolet Sunscreen Pigment
Source: Mar Drugs. 2020 Jun 4;18(6):300. doi: 10.3390/md18060300 (PMC7344946; doi:10.3390/md18060300)
Supplement: Supplementary file 1 [file marinedrugs-18-00300-s001.pdf]

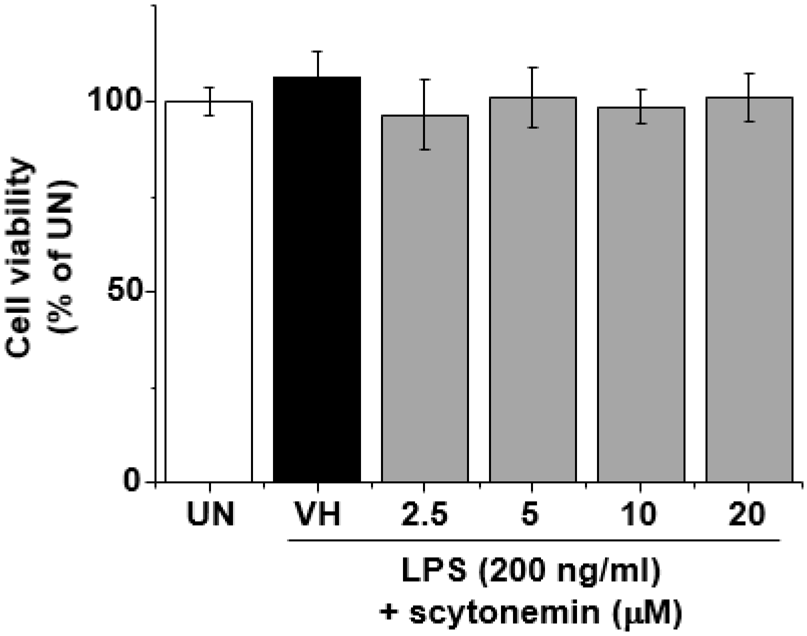

Supplementary Figure 1. Effect of scytonemin on the cell viability of LPS-stimulated RAW 264.7 cells. RAW 264.7 cells were pretreated with the indicated concentrations of scytonemin for 1 h before being incubated with LPS (200 ng/mL) for 24 h. Cell viability was measured by MTT assay. Each column shows the mean ± S.D. of triplicate determinations. Statistical significance was analyzed by one-way ANOVA and Dunnett's *t*-test (* $p < 0.05$).
